# Apolipoprotein A-I inhibited group II innate lymphoid cell response mediated by microRNA-155 in allergic rhinitis

**DOI:** 10.1016/j.jacig.2024.100212

**Published:** 2024-01-17

**Authors:** Yinhui Zeng, Qingxiang Zeng, Yueqiang Wen, Jinyuan Li, Haiqing Xiao, Chao Yang, Renzhong Luo, Wenlong Liu

**Affiliations:** a Department of Otolaryngology, Guangzhou Women and Children’s Medical Center, Guangzhou Medical University, Guangdong Provincial Clinical Research Center for Child Health, Guangzhou, China; b Department of Nephrology, the Second Affiliated Hospital, Guangzhou Medical University, Guangzhou, China

**Keywords:** Apolipoprotein A-I, group II innate lymphoid cells, microRNA-155, allergic rhinitis

## Abstract

**Background:**

Group 2 innate lymphoid cells (ILC2s) have been found to take part in type 2 inflammation by secreting T_H_2 cytokines. Apolipoprotein A-I (Apo-AI), a major structural and functional protein of high-density lipoproteins, has anti-inflammatory effects on neutrophils, monocytes, macrophages, and eosinophils. However, its effects on ILC2s are not well characterized.

**Objective:**

We aimed to investigate the effect of Apo-AI on the proliferation and function of ILC2s as well as its possible mechanism.

**Methods:**

The protein expression of Apo-AI and the percentage of ILC2s in peripheral blood between 20 allergic rhinitis patients and 20 controls were detected by ELISA and flow cytometry. The effect of Apo-AI and miR-155 on ILC2 proliferation and function was detected by tritiated thymidine incorporation and ELISA. Anima models were adopted to verify the effect of Apo-AI *in vivo*.

**Results:**

Elevated expression of Apo-AI was observed in allergic rhinitis patients. Apo-AI promotes *ABCA1* expression by ILC2s, which can be inhibited by anti–Apo-AI. Apo-AI decreased ILC2 proliferation and the microRNA levels of GATA3 and RORα from ILC2s. The miR-155 overexpression promoted the upregulation of GATA3 and type II cytokines from ILC2s, while the addition of Apo-AI or miR-155 inhibitor significantly inhibited expression of GATA3 and type II cytokines by ILC2s. Apo-AI^−/−^ mice showed as enhanced allergen-induced airway inflammation. The miR-155 inhibitor can reverse the enhanced allergen-induced airway inflammation in Apo-AI^−/−^ mice, while miR-155 mimics can reverse the decreased allergen-induced airway inflammation in Apo-AI–treated mice.

**Conclusion:**

Apo-AI suppressed the proliferation and function of ILC2s through miR-155 in allergic rhinitis. Our data provide new insights into the mechanism of allergen-induced airway inflammation.

Allergic rhinitis (AR) is a global issue with progressive incidence in recent years. In China, the standardized prevalence of AR of adult has increased by 6.5% over the last 6 years.^1^ AR is attributed to specific IgE-mediated inflammatory reaction against inhaled allergens and involves the activation of multiple immune cells.^2^

Apolipoprotein A-I (Apo-AI), synthesized by hepatocytes and enterocytes, is a major structural and functional protein of high-density lipoproteins in blood.^3^ Apo-AI interacts with its receptor, ATP-binding cassette transporter A1 (ABCA1), to facilitate the formation of high-density lipoprotein particles.^4^ Except for antiatherosclerotic properties, Apo-AI also plays anti-inflammatory effects on neutrophils, monocytes, macrophages, and eosinophils.^5^, ^6^, ^7^, ^8^ The effects of Apo-AI on asthma animal model had been reported in several studies.^9^, ^10^, ^11^ Apo-AI–deficient mice presented with aggravated lung inflammation compared to controls and treatment with Apo-AI mimetics alleviated airway inflammation in animal models.^9^, ^10^, ^11^ In detail, systemic treatment of 5A (Apo-AI mimetic peptide) significantly inhibited the infiltration of inflammatory cells in lung lavage fluid, while intranasal treatment of D-4F (Apo-AI mimetic peptide) exerted similar effect with systemic administration of 5A in ovalbumin (OVA) mouse model.^9^^,^^11^

Group 2 innate lymphoid cells (ILC2s) takes part in type 2 inflammation by secreting T_H_2 cytokines.^12^ ILC2s are generally activated by nasal mucosal epithelial cell–secreted cytokines, which include IL-33, IL-25, and thymic stromal lymphopoietin (TSLP).^13^ After activation, ILC2s produce and secrete T_H_2 cytokines.^14^ Theses cytokines promote eosinophil activation, mucus production, and IgE class switching. Therefore, innate and adaptive responses are linked.^14^

In AR, the numbers of circulating blood ILC2s seems to be related to allergen type. In house dust mite (HDM)-sensitized AR patients, serum IL-13 expression was positively correlated with elevated ILC2 frequency, but these changes were not observed in mugwort-sensitized AR patients.^15^ Moreover, number of ILC2s increased in pollen-sensitized AR subjects compared to controls.^16^ The frequency of peripheral blood ILC2s increased after cat allergen challenge.^17^

Although the role of ILC2s in AR has been preliminarily clarified, ILC2 regulation is not fully understood. In this study, we investigated the effect and possible mechanisms of Apo-AI on the regulation of ILC2s in AR by both *in vivo* and *in vitro* experiments.

## Methods

### Patient recruitment

Twenty AR patients allergic to HDM and 20 controls were recruited with the approval of the local ethical committee boards, and informed consent was obtained for all study subjects. The AR was diagnosed on the basis of typical symptoms (at least 1 year) and positive skin prick test result or finding of specific IgE to *Dermatophagoides pteronyssinus* and/or *Dermatophagoides farina,* as described previously.^18^ The exclusion criteria were as follows: pregnancy or breast-feeding, immunologic diseases, receipt of systemic or local corticosteroids in the last 4 weeks, history of smoking, and concomitant allergic disease such as asthma or atopic eczema. Asthmatic patients were excluded from the study by spirometric measurements. A total nasal symptom score ranging from 0 (not at all bothersome) to 3 (extremely bothersome) was summed to evaluated the severity of nasal symptoms. The 20 healthy controls had neither nasal symptoms nor positive allergen test results. Our study was approved by the local ethics committee (approval 261A01).

### ILC2 sorting and culture

Peripheral blood mononuclear cells (10 mL) were obtained from peripheral blood of AR patients using density gradient centrifugation. Cell viability (>98%) was confirmed by trypan blue exclusion. ILC2s were stained and sorted by a Beckman flow cytometer and a MoFlo XDP cell sorter (Beckman Coulter). The following antibodies were used: fluorescein isothiocyanate–conjugated lineage markers (CD4, CD3, CD14, CD16, CD235a, CD19, CD20, FcεRIα, TCR-γ/δ, CD56), allophycocyanin-conjugated CD161, phycoerythrin (PE)-conjugated CRTH2, PE-Cy7–conjugated CD127, and PE-Cy7–conjugated ABCA1. Sorted ILC2s (1 × 10^4^ cells/mL, >98% purity) were cultured in media that added recombinant human IL-2 (10 ng/mL), recombinant human IL-7 (40 ng/mL), IL-33 (50 ng/mL), or HDM (1.6 μg/mL) for 9 days.

For intracellular staining of IL-5 and IL-13, ILC2s were first activated by phorbol 12-myristate 13-acetate (aka PMA; 10 ng/mL; Sigma-Aldrich) and ionomycin (500 nmol; Merck) for 3 hours at 37°C and stained with fluorescein isothiocyanate–conjugated IL-5 and PE-conjugated IL-13. ILC2 proliferation was detected by tritiated thymidine incorporation.

### Western blot analysis

Total proteins were extracted from ILC2s with cold radioimmunoprecipitation assay buffer. Proteins were quantified by bicinchoninic acid assay (Thermo Fisher Scientific), separated by gel electrophoresis, and transferred to a polyvinyl difluoride membrane. The membranes were incubated overnight with JAK, p-JAK, MAPK, p-MAPK, ERK, p-ERK, or β-actin (1:1000; Santa Cruz Biotechnology) overnight at 4°C. After a second wash with Tris-buffered saline, the secondary antibodies were used for 1 hour. The bands were detected by electrochemiluminescence Western blot substrate (Thermo Fisher Scientific) and quantified by ImageJ software (imagej.nih.gov/ij).

### Quantitative real-time PCR

Total RNA was extracted by a NucleoSpin RNA XS kit (Macherey-Nagel). The cDNA was synthesized by Oligo(dT) (12-18 mers) primer with SuperScript II as the reverse transcriptase. PCR was done with the ABI PRISM 7300 Detection System. MicroRNA (miRNA) levels were normalized to expression of the housekeeping gene. Primers were as follows: GATA3 sense, 5′-GCGGGCTCTATCACAAAATGA-3′, antisense, 5′-GCTCTCCTGGCTGCAGACAGC-3′; retinoic acid receptor–related orphan receptor α (RORα) sense, 5′-AAGGAGCCAGAAGGGATGAAC-3′, antisense, 5′-GGAACA ACAGACGCCAGTAAG-3′; ABCA1 sense, 5′-AGGAAACCCAATCCCAGATACCC-3′, antisense, 5′-GCTCGGAGGAAGTGCTTGAGAAT-3′; IL-5 sense, 5′-GCTTCTGCATTTGAGTTTGCTAGCT-3′, antisense, 5′-TGGCCGTCAATGTATTTCTTTATTAAG-3′; IL-13 sense, 5′-CCCAGAACCAGAAAGGCTCCG-3′, antisense, 5′-CAGTTGAACCGTCCCTGCCG-3′; β-actin sense, 5′-CGAAACTACCTTCAACTCCATC-3′, antisense, 5′-AGTGATCTCCTTCTGCATCCT-3′.

### Enzyme-linked immunosorbent assay

Cytokine expression was determined by ELISA kits (R&D Systems). The detection limits were as follows: Apo-AI, 6.3 ng/mL; IL-5, 3.9 pg/mL; IL-13, 125 pg/mL; IL-4, 1.56 pg/mL; IL-25, 11.7 pg/mL; TSLP, 9.87 pg/mL; and IL-33, 1.51 pg/mL. The detection of OVA-specific IgE was performed by ELISA kits (Cusabio Technology).

### Human nasal epithelial cell culture and treatment

Human nasal epithelial cells (HNECs) were purchased and cultured using Airway Epithelial Cell Growth Medium (PromoCell) at 37°C and 5% CO_2_. HNECs were cultured under stimulation of 10-100 ng/mL Apo-AI and 1.6 μg/mL of *D pteronyssinus,* and the supernatant was detected after 24 hours.

### Mouse model

Eight-week-old female BALB/c or Apo-AI^−/−^ mice were immunized by intraperitoneal injection of 100 μg of OVA and 1.6 mg Al(OH)_3_ in PBS on day 0 and day 7. The intranasal challenge was performed by 100 μg of OVA (+10 mg/kg Apo-AI in some mice) on days 14, 16, and 19. The mice were humanely killed after the last challenge. Nasal and blood samples were obtained for further analysis. All animal handling protocols received approval from local animal ethics committee boards. Hematoxylin and eosin staining was done as previously described.^19^^,^^20^

### Isolation of mouse ILC2s

Single-cell suspensions from nasal tissue and blood were prepared and enriched by Percoll gradient centrifugation. Lineage-negative cells were further enriched using autoMACS (Miltenyi Biotec). Then the percentage of Lin^−^CD45^+^ST2^+^ ILC2s was determined by flow cytometry. IL-5– and IL-13–positive ILC2s were assayed by intracellular cytokine staining as described above.

### Statistical analysis

Statistical analysis was performed by GraphPad Prism 9 (GraphPad Software). Data are shown as means ± standard errors of the mean. Comparisons among groups were done by Mann-Whitney *U* test, 1-way ANOVA, or Student *t* test. Correlation analysis was done by the Spearman rank method. *P* < .05 was considered statistically significant.

## Results

### Serum protein concentration of Apo-AI and its relation with percentage of ILC2s in AR

**Fig 1:**
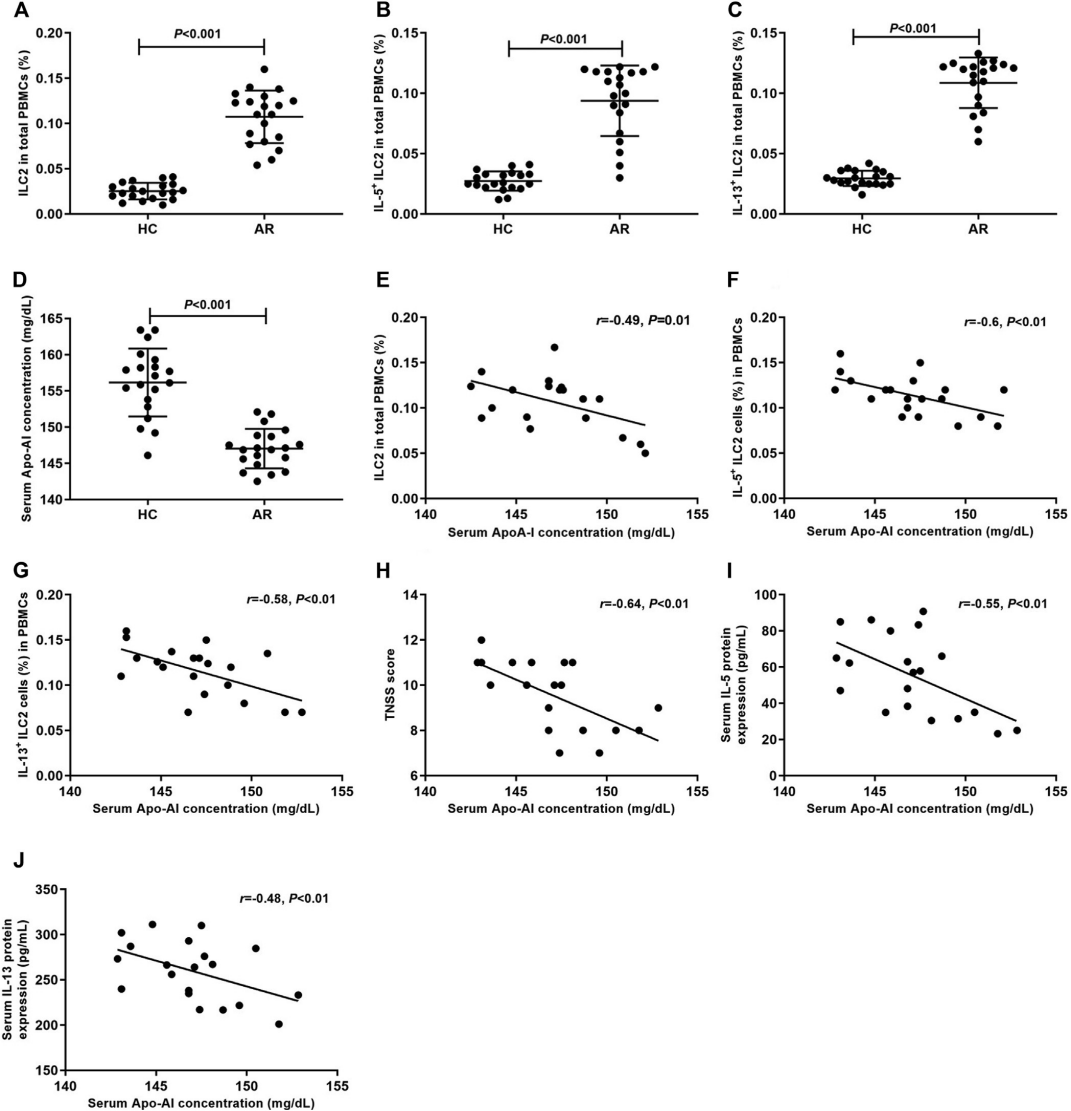
Serum level of Apo-AI protein in AR and its correlation with percentage of ILC2s. **(A-C)** Percentage of ILC2s, IL-5^+^ ILC2s, and IL-13^+^ ILC2s in PBMCs between AR and controls detected by flow cytometry. **(D)** Serum level of Apo-AI protein in AR and controls by ELISA. **(E-H)** Correlation between Apo-AI protein and percentage of ILC2s, IL-5^+^ ILC2s, IL-13^+^ ILC2s, and TNSS. (**I** and **J**) Correlation between Apo-AI protein and serum protein of IL-5 and IL-13. *HC,* Healthy control; *PBMC,* peripheral blood mononuclear cell; *TNSS,* total nasal symptom score.

Basic information of AR and controls is listed in Table I. The percentage of ILC2s, IL-5^+^ ILC2s, and IL-13^+^ ILC2s in peripheral blood mononuclear cells in AR was significantly higher than in controls (*P* < .01) (Fig 1, *A-C*). The serum level of Apo-AI protein in AR was significantly downregulated compared to controls (*P* < .01) (Fig 1, *D*). The serum level of Apo-AI protein in AR patients was negatively related to percentages of ILC2s, IL-5^+^ ILC2s, IL-13^+^ ILC2s, and total nasal symptom score (Fig 1, *E-H*). The serum level of Apo-AI protein in AR patients was also negatively related to serum levels of IL-5 and IL-13 protein (Fig 1, *I* and *J*).

**Table I:** Demographic characteristic of AR children and controls

Characteristic	AR	Control
No. of subjects	20	20
Sex (M:F)	12:8	11:9
Age (months), mean ± SEM	24.1 ± 5.7	22.6 ± 4.6
Symptom duration (years), mean ± SEM	2.1 ± 1.7	—
Serum IgE level to Der p (IU/mL)	23.1 (5.8-98.4)	—
Serum IgE level to Der f (IU/mL)	34.7 (3.1-78.3)	—
Total IgE (IU/mL)	365.3 (89.1-1124.3)∗	98.1 (33.2-743.8)

∗ Statistically significant compared to control group, *P* < .05.

### ILC2 proliferation and function regulated by Apo-AI mediated by miR-155

**Fig 2:**
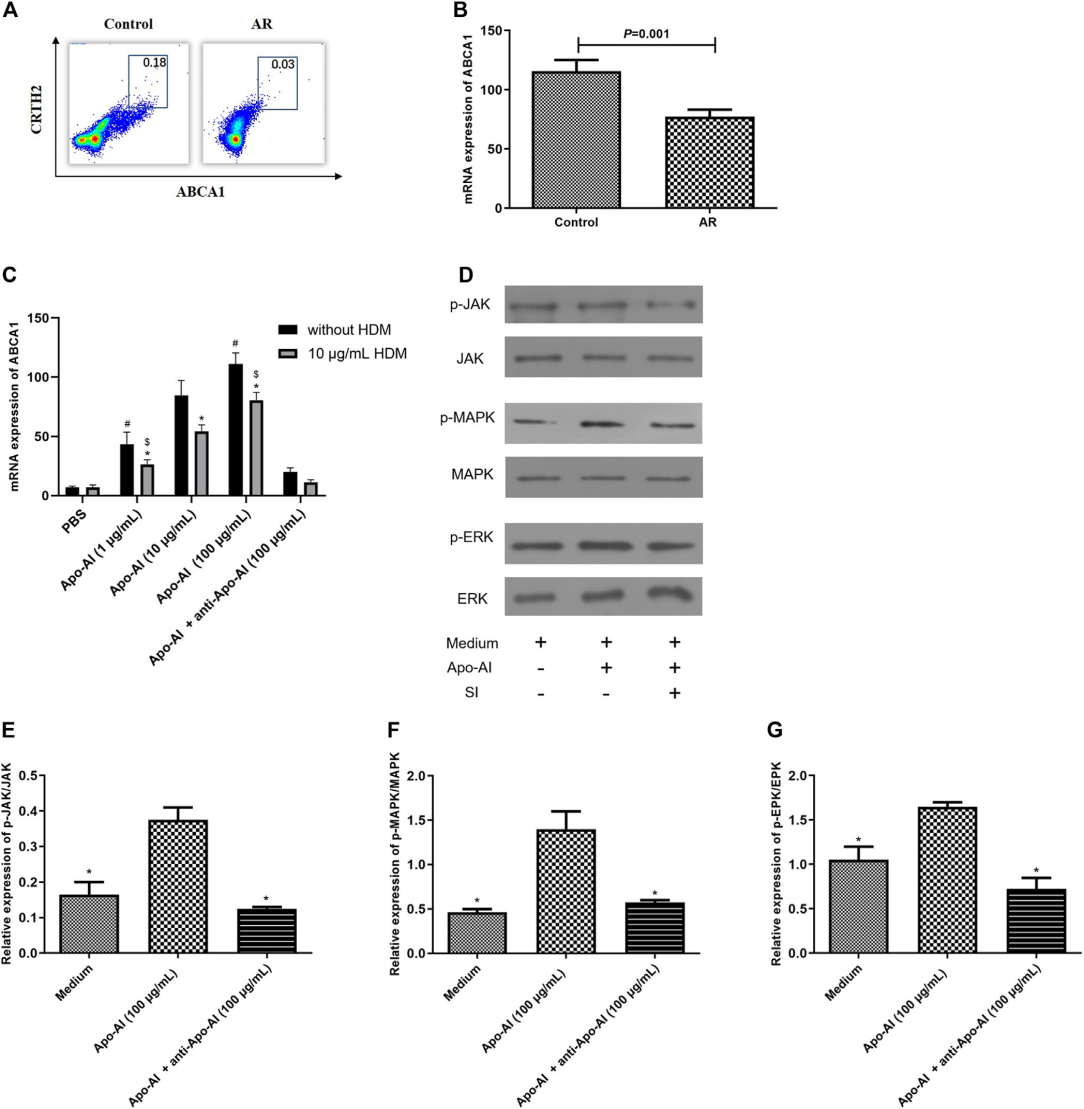
miRNA expression of *ABCA1* by ILC2s and related signal pathways. (**A** and **B**) Surface and miRNA expression of *ABCA1* by ILC2s between AR and controls detected by flow cytometry and RT-PCR. **(C)** miRNA expression of *ABCA1* by ILC2s regulated by Apo-AI detected by RT-PCR. **(D-G)** Phosphorylation of JAK, MAPK, and ERK detected by Western blot and their relative expression. *(C)* ∗Compared to without HDM group, *P* < .05; #compared to 10 μg/mL group (without HDM), *P* < .05; $compared to 10 μg/mL group (HDM), *P* < .05. *(E-G)* ∗Compared to Apo-AI group, *P* < .05. *RT-PCR,* Real-time reverse transcriptase PCR.

The surface and miRNA expression of *ABCA1* by ILC2s in controls was significantly higher than in AR patients (Fig 2, *A* and *B*). Apo-AI promotes *ABCA1* expression by ILC2s and can be inhibited by anti–Apo-AI (Fig 2, *C*). Moreover, phosphorylation of JAK, MAPK, and ERK was found in ILC2s stimulated by Apo-AI (Fig 2, *D-G*).

**Fig 3:**
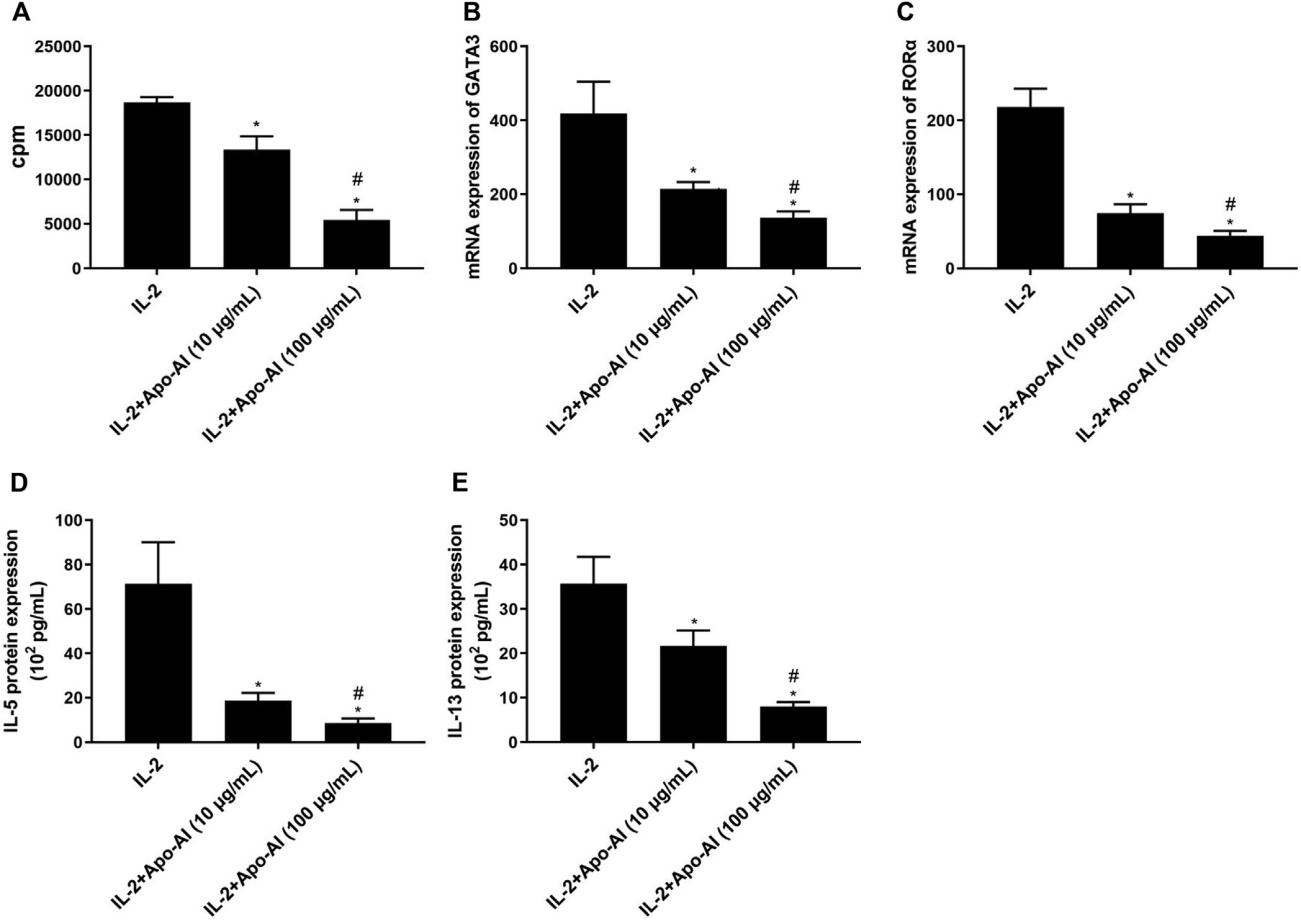
miRNA expression of *ABCA1* by ILC2s and related signal pathways. **(A)** Proliferation of ILC2s regulated by Apo-AI. (**B** and **C**) miRNA levels of GATA3 and RORα from ILC2s regulated by Apo-AI detected by RT-PCR. (**D** and **E**) Protein expression of IL-5 and IL-13 from ILC2s regulated by Apo-AI detected by ELISA. ∗Compared to IL-2 group, *P* < .05. #Compared to 10 μg/mL group, *P* < .05. *RT-PCR,* Real-time reverse transcriptase PCR.

Apo-AI decreased the proliferation of ILC2s and the miRNA levels of GATA3 and RORα from ILC2s (Fig 3, *A-C*). After Apo-AI stimulation, the protein expression of IL-5 and IL-13 from the supernatant of ILC2s was downregulated compared to controls (Fig 3, *D* and *E*). However, IL-4 protein expression was undetectable.

**Fig 4:**
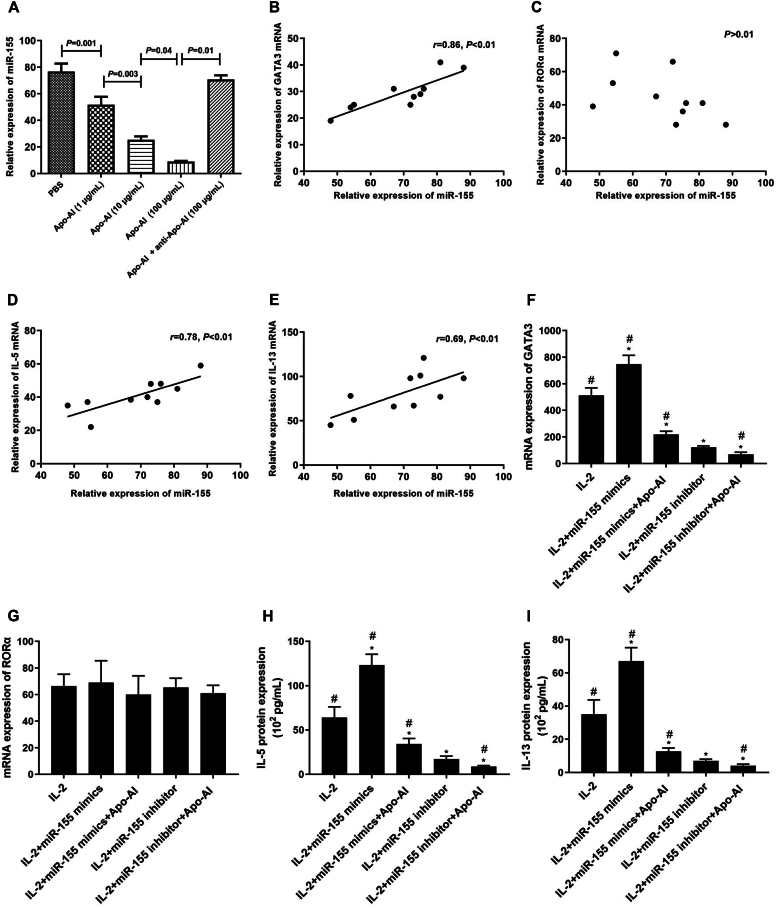
Regulation of ILC2s by Apo-AI mediated through miR-155. **(A)** Expression of miR-155 in ILC2s regulated by Apo-AI. **(B-E)** Correlation between miR-155 expression and GATA3, RORα, IL-5, and IL-13, respectively. (**F** and **G**) Effect of miR-155 inhibitor or mimics in regulation of GATA3 and RORα expression. (**H** and **I**) Effect of miR-155 inhibitor or mimics in regulation of ILC2 function. ∗Compared to IL-2 group, *P* < .05. #Compared to miR-155 inhibitor group, *P* < .05.

Apo-AI inhibited the expression of miR-155 in ILC2s (Fig 4, *A*), and the expression of miR-155 was positively related to the expression of GATA3, IL-5, and IL-13 rather than RORα (Fig 4, *B-E*). We also found that miR-155 overexpression promoted upregulation of GATA3, IL-5, and IL-13 from ILC2s, while the addition of Apo-AI or miR-155 inhibitor significantly downregulated the levels of GATA3, IL-5, and IL-13 from ILC2s (Fig 4, *F, H,* and *I*). Moreover, Apo-AI can enhance the ability of miR-155 inhibitor in limiting ILC2 function (Fig 4, *F, H,* and *I*). However, RORα expression was not affected by miR-155 (Fig 4, *G*).

### Effect of Apo-AI on expression of ILC2 differentiation factor by HNECs

**Fig 5:**
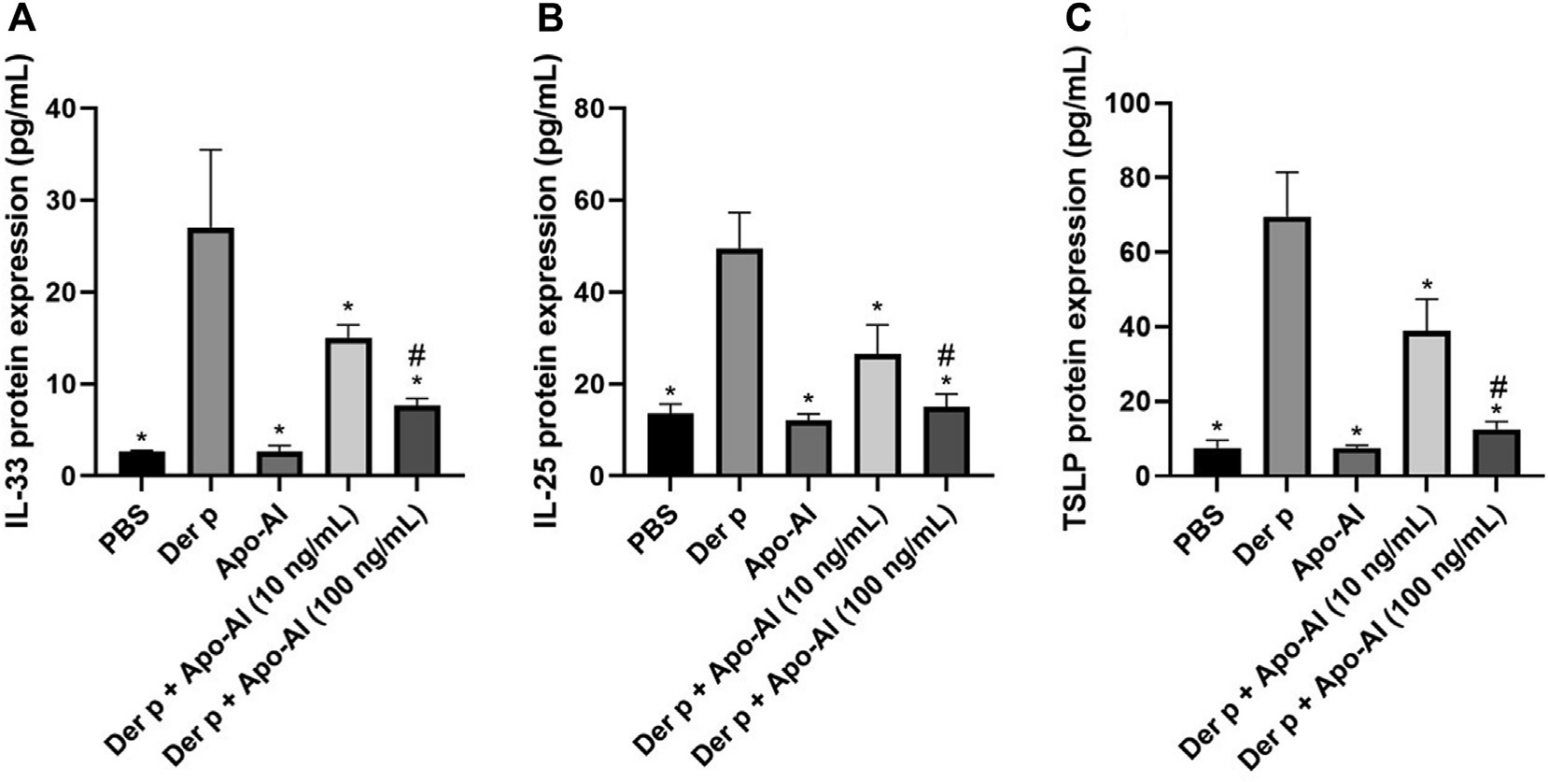
Proinflammatory cytokines released from HNECs after treated with Apo-AI. **(A-C)** Protein levels of, respectively, IL-25, IL-33, and TSLP secreted from Apo-AI (10-100 ng/mL)- or *Dermatophagoides pteronyssinus* (Der p, 1.6 μg/mL)-treated HNECs. ∗Compared to Der p group, *P* < .05. #Compared to 10 ng/mL group, *P* < .05.

After *D pteronyssinus* stimulation, the protein levels of IL-25, IL-33, and TSLP secreted from HNECs were significantly upregulated compared to PBS-treated controls. When Apo-AI (10-100 ng/mL) was added, their expression levels decreased significantly (Fig 5).

### Apo-AI inhibits ILC2 responses in mice

**Fig 6:**
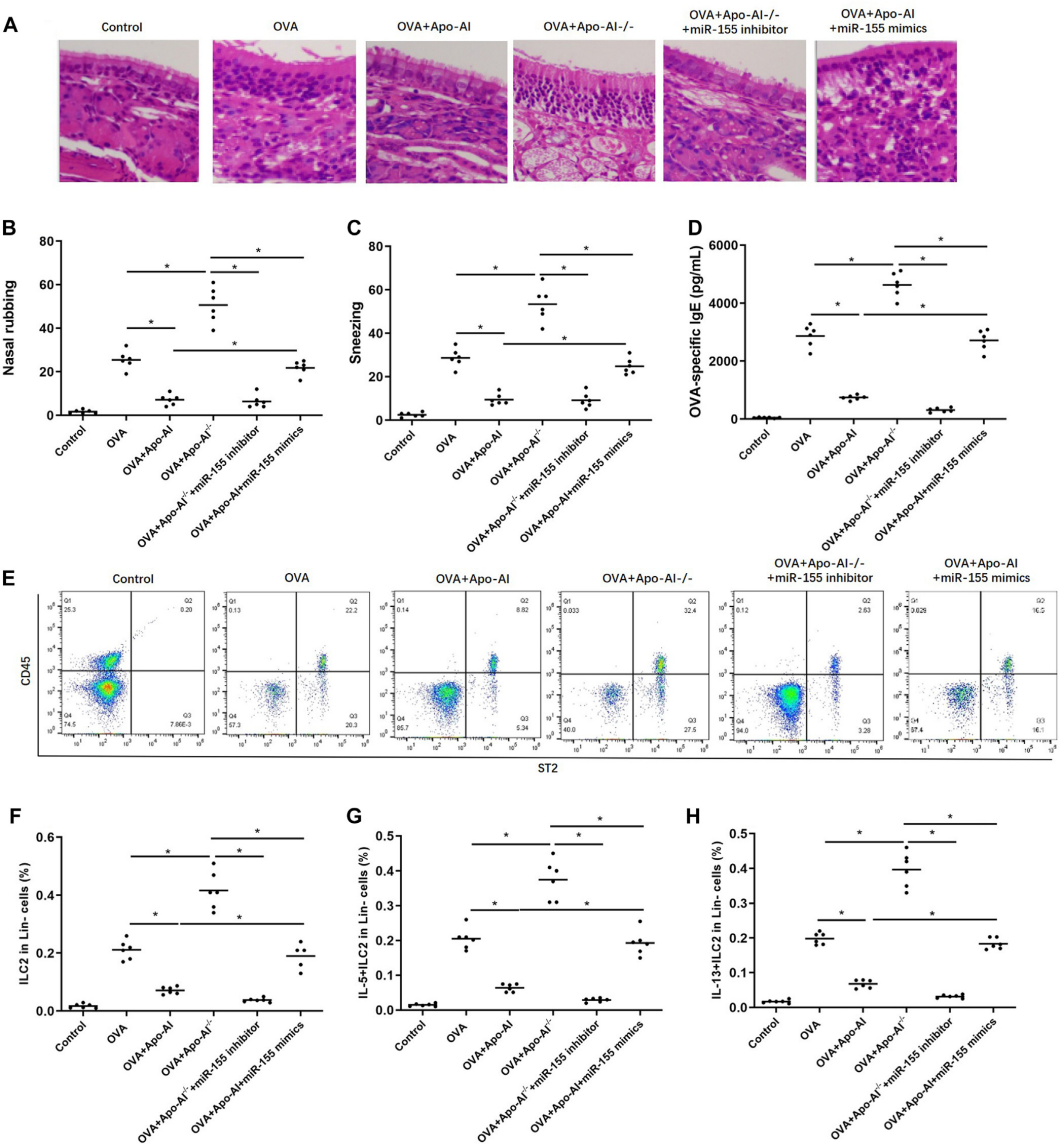
Regulation of ILC2s by Apo-AI in mouse model. **(A)** Hematoxylin and eosin staining of nasal section in different groups. (**B** and **C**) Nasal symptoms of mice in different groups. **(D)** Serum OVA-specific IgE levels in different groups. **(E-H)** Percentage of ILC2s, IL-5^+^ ILC2s, and IL-13^+^ ILC2s between different groups. ∗Compared to groups, *P* < .05.

In Apo-AI–treated mice, the number of eosinophils and the thickness of epithelium, the frequency of nasal rubbing and sneezing, and the levels of OVA-specific IgE decreased significantly compared to OVA-treated mice, while these markers elevated significantly in OVA-treated Apo-AI^−/−^ mice (Fig 6, *A-D*). Consistently, Apo-AI–treated mice presented with a lower percentage of ILC2s, IL-5^+^ ILC2s, and IL-13^+^ ILC2s than did OVA-treated mice, while these markers increased significantly in OVA-treated Apo-AI^−/−^ mice (Fig 6, *E-H*). Moreover, the miR-155 inhibitor can reverse the enhanced allergen-induced airway inflammation in Apo-AI^−/−^ mice, while miR-155 mimics can reverse the decreased allergen-induced airway inflammation in Apo-AI–treated mice (Fig 6).

## Discussion

The key roles of ILC2s in allergic inflammation have been investigated recently, and increasing evidence suggests that its regulation involves various cytokines and signal pathways. Apo-AI plays a protective role in airway allergic inflammation. However, its effects on ILC2s were not clear. In this study, we showed that Apo-AI suppressed the proliferation and function of ILC2s through miR-155 in AR.

In proteome studies, Apo-AI levels in AR have been found to be significantly higher compared to controls.^21^^,^^22^ Another study also suggested a positive correlation between plasma Apo-AI concentrations and prevalence of wheeze and asthma.^23^ However, Trakaki et al^24^ showed that Apo-AI was significantly downregulated in AR compared to controls. Moreover, Cirillo et al^25^ reported that serum Apo-AI levels were negatively linked to airflow obstruction in asthmatic patients. Our data suggest that the serum Apo-AI protein in AR is significantly downregulated compared to controls as well as negatively related to ILC2 number and function.

To confirm the direct effect Apo-AI on ILC2s in AR, we stimulated ILC2s with Apo-AI. We found that Apo-AI regulated the expression of its receptor, *ABCA1,* by ILC2s through the phosphorylation of JAK, MAPK, and ERK. ILC2 proliferation, transcription factor GATA3 and RORα, and type II cytokine production could be significantly inhibited by Apo-AI. These results suggest that Apo-AI can directly affect the proliferation and function of ILC2s.

miR-155 plays a central role in allergic inflammatory response.^26^ Johansson et al^27^ found that miR-155 regulates ILC2s via IL-33 signaling. They found that IL-33–challenged miR-155^−/−^ mice presented with decreased ILC2 proliferation and function compared to IL-33–challenged wild-type ILC2s. Consistently, we also confirmed that miR-155 is necessary in Apo-AI–mediated ILC2 regulation because the proliferation and function of ILC2s were not affected when miR-155 was knocked down.

ILC2 activation depends on the IL-25, IL-33, and TSLP secreted from HNECs. Thus, we investigated the effect of Apo-AI on the production of IL-25, IL-33, and TSLP. As expected, Apo-AI inhibited the expression of these cytokines, especially IL-33, which is the only necessary cytokine for an intact allergic immune response.

In murine models, we found that Apo-AI^−/−^ mice showed enhanced allergen-induced airway inflammation. Interestingly, the miR-155 inhibitor can reverse the enhanced allergen-induced airway inflammation in Apo-AI^−/−^ mice, while miR-155 mimics can reverse the decreased allergen-induced airway inflammation in Apo-AI–treated mice, again suggesting a central effect of miR-155 in allergic inflammatory response.

In this study, we found that Apo-AI suppressed the proliferation and function of ILC2s through miR-155 in AR. Our data provide new insights into the mechanism of allergen-induced airway inflammation.

## Disclosure statement

Supported by a National Natural Science Grant of China (grants 82271142 and 81970861), a Guangdong Province Natural Science grant (grant 2021A1515010940), and the Science and Technology Program of Guangzhou (grants 202201020600 and 202201011844), and the Scientific Research Capacity Improvement Project of Guangzhou Medical University (02-410-2302151XM).

Disclosure of potential conflict of interest: The authors declare that they have no relevant conflicts of interest.
